# On the Path to Thermo-Stable Collagen: Culturing the Versatile Sponge *Chondrosia reniformis*

**DOI:** 10.3390/md19120669

**Published:** 2021-11-26

**Authors:** Boaz Orel, Marco Giovine, Micha Ilan

**Affiliations:** 1 School of Zoology, George S. Wise Faculty of Life Sciences, Tel Aviv University, Ramat Aviv, Tel Aviv 6997801, Israel; milan@tauex.tau.ac.il; 2 Department of Sciences of Earth, Environment and Life, University of Genoa, Corso Europa 26, 16132 Genoa, Italy; mgiovine@unige.it

**Keywords:** Porifera, culture, collagen, temperature effect, transplantation, mesophotic, Levant

## Abstract

The collagen proteins family is sought-after in the pharmaceuticals, cosmetics, and food industries for various biotechnological applications. The most abundant sources of collagen are pigs and cows, but due to religious restrictions and possible disease transmission, they became less attractive. An alternative source can be found in marine invertebrates, specifically in sponges. Alas, two problems arise: (1). Growing sponges is complicated. (2). Sponge collagen has low heat tolerance, which can impose a problem for human biotechnological usage. To fill these gaps, we studied the collagen-abundant sponge *Chondrosia reniformis*. Two culture experiments were conducted: (1). A sea-based system examined the difference in growth rates of *C. reniformis* from different habitats, growing under natural seasonal conditions; (2). A land-based controlled system, which assessed the growth-rate of *C. reniformis* at different temperatures. The results reveal that *C. reniformis* from shallow habitats are growing larger and faster than individuals from colder, deeper habitats, and that the optimal temperature for *C. reniformis* growth is 25 °C. The results demonstrate that *C. reniformis* is highly fit for culture and can produce thermally stable collagen. Further research is needed to determine the best conditions for *C. reniformis* culture for collagen extract and other exciting materials for bioprospecting.

## 1. Introduction

The main organic component of the metazoan extracellular matrix is made of the collagen family of proteins [1]. Collagens appear in various organizational forms, and carry many mechanical and physiological functions [2]. Collagen, therefore, has many potential applications in tissue engineering, drug delivery, fracture recovery, and the cosmetic and food industries. Thus, the collagen market is growing fast worldwide. In 2016, its value was estimated at 3.61 billion dollars, and its expected value in 2025 was estimated at 6.63 billion [3]. However, one of the major difficulties in collagen manufacture is a supply problem. For years, cows and pigs were the primary sources for collagen production [4]. In the last decade, those sources became limited for two main reasons: (1) religious restrictions (Judaism, Buddhism, Hinduism, and Islam); (2) fear of transmission of zoonotic diseases such as foot-and-mouth disease and bovine spongiform encephalopathy [5]. Therefore, a demand for new sources of collagen has emerged.

Marine-derived collagen has been suggested as another source for use. Two recent reviews reported of different types of collagens (I, II, IV, V, and XI) extracted from marine mammals (Minke whale), through fish, to various invertebrates [6,7]. They further showed that these collagens were proposed to be used in multiple applications, depending on the type of collagen [6]. Marine invertebrates are recognized as an emerging alternative source of collagen. Their collagen is preferable for several reasons: it has better water absorption; it does not stimulate inflammatory and rejection reactions; it scarcely contains any biological pollution (diseases); it is also more ethically acceptable [8,9]. In addition, collagen from marine invertebrates is produced with relative ease, high efficiency, and low cost for industrial and medical applications [10]. Sponges (phylum Porifera), the oldest metazoans living on our planet, are a rich source of collagen among the marine invertebrates, since, in many cases, their skeleton and extracellular matrix are made mostly from collagen [11,12]. Sponges are filter-feeding organisms that inhabit all aquatic habitats [13]. More than 9000 species are known today [14], divided into four classes. They also constitute a major source for numerous (marine-derived) natural products [15], many of which have the potential for development into pharmaceutical, cosmeceutical, or agricultural products [16,17]. However, there are two potential obstacles in the industrial/large-scale production of collagen from sponges: (1) Since sponges (as with all marine invertebrates) are poikilothermic and live in temperatures much lower than the human body, sponge-derived collagen usually has a lower thermal stability compared to the that of homeothermic mammals and birds [8,18,19]. This raises the question as to whether sponge-derived collagen is suitable for use in humans. (2) Despite the ever-growing demand for sponge-originated natural products, there are neither sustainable and economic culture methods to deliver large and stable biomasses of sponges [20,21], nor is there a cost-effective method to chemically synthesize many of the sponge-derived natural products [20]. 

The Mediterranean marine sponge *Chondrosia reniformis* (Nardo, 1847, Demospongiae) has a skeleton made of high amount of collagen fibers [12] without any inorganic component (spicules). In addition to collagen, this species contains various compounds with pharmaceutical implications [22,23]. Its fast regeneration and its asexual reproduction abilities, as well as the capability to selectively incorporate minerals into its collagenous skeleton, make this species exceptionally resilient [24,25,26] and amenable for manipulations. Moreover, the collagen of *C. reniformis* is the most studied one among the phylum Porifera, and it has been biochemically and physiologically partly characterized [27,28,29]. It was shown that collagen from *C. reniformis* is effective for wound healing, and also for its antioxidant activity [30,31]. For these reasons, *C. reniformis* was the target of several aquaculture studies [32,33,34] aiming at acquiring large amount of collagen for further industrial processing. It is no wonder that a recent review about sponge collagen paid special attention to *Chondrosia* sp. collagens [35]. In the eastern Mediterranean (Levant) Sea, *C. reniformis* grows from the shallow water to the mesophotic depth, where the water temperature ranges are (17–31 °C) and (16–20 °C), respectively. We aimed at developing new ways of culturing specimens of *C. reniformis* from both habitats: the relatively cool mesophotic depth (~100 m) and the much warmer shallow (~3–5 m) water. The hypotheses were: (1) sponges collected from shallow depth (a less stable environment) will be less sensitive to human handling; (2) providing sponges with 3-micron filtered running seawater (supplemented with food) will result in high survivorship and growth, which will yield large amounts of collagen; (3) shallow water sponges that naturally thrive through extended periods of high temperature will contain collagen with higher thermal stability, which is better fit for human use. 

## 2. Results

### 2.1. Sea-Based Mariculture System Experiment

**Figure 1:**
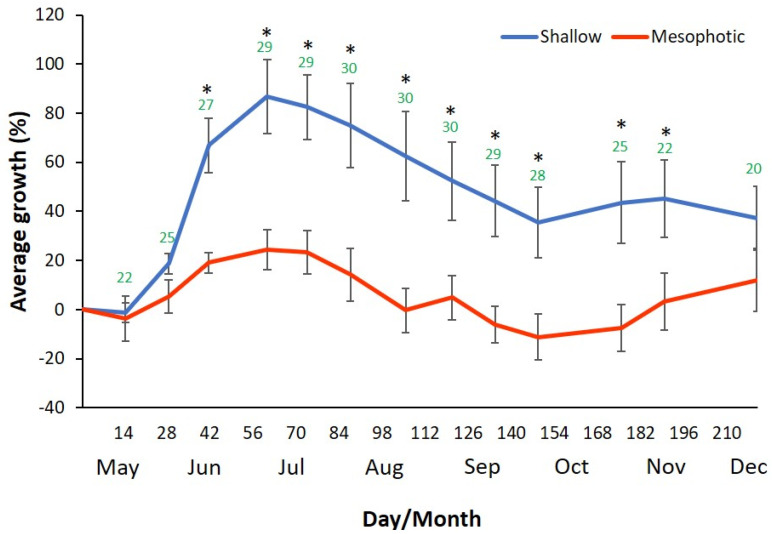
Relative growth rate percentage of *C. reniformis* in the sea-based mariculture system experiment. The slope represents the growth rate of the sponges relative to the start of the experiment. The experiment was conducted between May 2019 and January 2020. Orange line = mesophotic habitat sponges (*n* = 15). Blue line = shallow habitat sponges (*n* = 15). Asterisk = statistically significant difference between groups (Mann–Whitney *p* < 0.05). Green numbers = water temperature in Celsius.

The growth of sponges from shallow and mesophotic habitats was first examined in a sea-based mariculture system experiment, with 15 specimens in each group divided into five cages. The size of every individual was measured to calculate percent growth, and the mean growth of all the individuals was examined at the same time (Figure 1). All sponges survived the transplantation and the entire duration of the experiment (210 days). The sponges that originated from both shallow and mesophotic depth showed similar growth patterns: they grew in the spring and fall (up to 86% and 24% in the shallow and mesophotic groups, respectively), and shrank in the summer and winter (down to 40% and −10% in the shallow and mesophotic groups, respectively). However, the mean percent growth of sponges from the shallow habitat was significantly higher than the mesophotic sponges.

Moreover, the shallow habitat group grew faster and larger. In addition, towards the end of the experiment the mesophotic sponges showed some stress signs (milky texture and color). In Figure 1, the slopes of the curves indicate the mean growth rates of the two groups. The sponges collected from the shallow habitat had grown faster and reached + 86% average growth after 60 days, during which the mesophotic sponges reached only + 24% average growth. The steepest slope, which indicates the fastest growth rate, was between day 28 and day 42 of the experiment, both in the shallow and the mesophotic group. The ambient seawater temperature at that time was 25–27 °C. *p*-values of the mean growth rate differences between the groups and water temperature are listed in Table A1 and Table A2.

### 2.2. Land-Based Culture System Experiment

**Figure 2:**
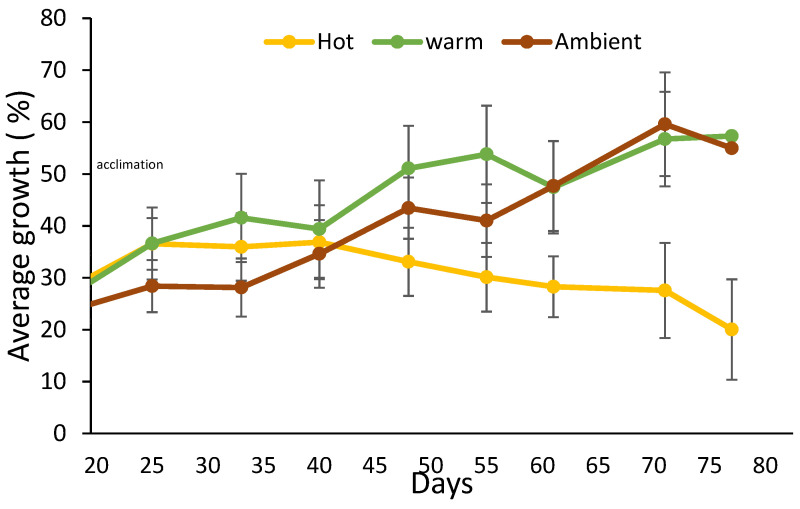
Relative growth percentage of *C. reniformis* at different temperature treatments in land-based mariculture system experiment. Yellow line = 31 °C (Hot) treatment; green line = 25 °C (Warm) treatment; Brown line = Mediterranean Sea ambient water temperature rose from 18 to 25 °C (warm) during the experiment. (*n* = 12 for each treatment).

**Figure 3:**
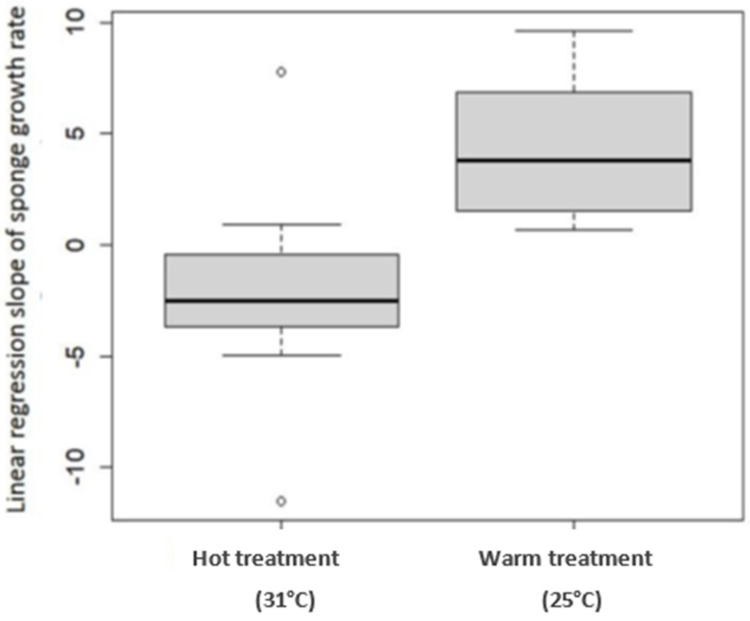
Linear regression slope of sponge growth rate at Hot and Warm temperature treatments. Box plots for 31 °C and 25 °C water temperature treatments (*n* = 12 in each treatment). The growth rate at 25 °C was significantly higher than in 31 °C (two-sample *t*-test, *t* = −4.2624, *p*-value = 0.0005).

All sponges survived the transplantation to the land-based experimental setup, and the entire experiment duration (91 days). The sponges’ size measurement started at the end of an acclimatization period—day 0. However, the statistical analysis performed on the data was from day 25 of the experiment onwards, since on the 25th day, the treatment reached the desired water temperature. Until then, all the sponges grew (therefore, in Figure 2, the curve does not start from 0%). In the 25 °C (Warm) treatment, from day 25, the slope of the curve was positive, reflecting sponge growth until reaching a peak of 57% growth on day 77 of the experiment. In the 31 °C (Hot) treatment, from day 40, a negative slope was observed since the sponges shrank until they reached a negative 20% growth, relative to their size at the beginning of the experiment. By day 60 of the experiment, the sponges in the ambient treatment grew the same as the sponges in the Warm treatment. This coincided with a constant increase in the ambient seawater temperature from 18 to 25 °C (reaching the temperature of Warm treatment). Figure 3 shows the regression slopes of the growth rates in the Warm and the Hot treatments. The (positive) slope of the Warm treatment is much larger than the Hot treatment (negative) slope (*p*-value = 0.0005), i.e., a higher growth rate is indicated.

## 3. Discussion

It is accepted that mariculture in general and sponge culture in particular should be “green”, sustainable, and economical. This work examined sea-based and land-based sponge mariculture methods for acquiring collagen of high biotechnological value from the collagen-rich sponge *C. reniformis*. We first checked the effect of water temperature on the sponge growth rate (to find the optimal conditions for sponge culture), aiming to obtain thermally stable collagen.

All the sponge specimens collected for this study survived for the entire duration of the experiments, both in marine-based and land-based experimental setups. This is in contrast to many sponge culture studies that showed a decline in survivorship in experiments involving transplantation [36,37,38,39]. The relatively small number of successful sponge cultures is probably related to the diversity in ecological and physiological characters of species within the phylum (Porifera). Thus, some of these characters are related to the non-optimal environmental conditions at the chosen culture sites [40,41]. 

The unique “movement” ability of *Chondrosia reniformis* has made it infamous as a problematic species for sea-based mariculture since it can “run away” from its designated place through branching, shrinking, and asexual reproduction ability. To overcome such phenomena, in the current research, the sponges were transplanted to the sea-based location in plastic cages that were connected to the rocky terrain and, therefore, the sponges did not “escape”. In addition, both mesophotic and shallow water *C. reniformis* specimens were successfully transferred to the sea-based culture system (100% survival). 

In the sea-based experiment, the sponges that originated in a shallow habitat grew significantly faster than those originated in the mesophotic habitat. Throughout the experiment, the shallow group grew (growth peak of 86%) faster than the mesophotic group (growth peak of 20%). The latter group even showed some stress signs. Following acclimation, all sponges in the sea-based experiment grew while the ambient temperatures increased to 28–29 °C, and higher temperatures were detrimental. Despite the difference in growth rate between the two groups, the sponges from both habitats grew (in the spring) or shrank (in mid-summer and winter) simultaneously. These observations indicate a trend of seasonality that is possibly related to changes in water temperature, a conclusion that correlates with other studies [42]. Other studies that tested the effect of temperature and seasonality on sponge growth showed confusing and contradictory results [43,44]. The difference in growth rate suggests a high-temperature adaptation of the shallow habitat *C. reniformis* group. It was previously demonstrated that for sponges from the same species that grow in different habitats, an environmental adaptation process can occur. Such adaptational processes could lead to genetic differentiation and, thus, ecologically derived speciation [45]. Maybe such adaptation led the shallow habitat’s *C. reniformis* to better cope with the extreme high-water temperature of the Israeli summer, which opens the possibility for its collagen to be more stable at high temperature and better suited for medical and cosmetic uses than collagen biosynthesized at colder temperatures (i.e., in mesophotic specimens). Indeed, the examination of collagen from both mesophotic and shallow water specimens showed higher thermal stability of collagen from shallow water sponges (Tassara et al., manuscript in preparation). This sea-based culture experiment indicates the importance of targeting and selecting a suitable sponge population for culture to obtain high-value (thermally-stable) collagen that is preferable for cosmetics, but not only cosmetics [46]. 

To better evaluate the effects of temperature (31 °C, 25 °C, and 18 °C) on the growth of *C. reniformis*, a land-based culture system was built. In this system, sponges were successfully cultured for three months (100% survival). The sponges of the Warm and Ambient treatments flourished (grew and did not show any sign of disease) for the entire duration of the experiment. This result opens many possibilities for inland *C. reniformis* culture and experiments. There are many diverse applications for sponge culture in controlled land-based systems, such as: sponge culture for harvesting natural and biological products, filtering organic pollutants from aquatic systems, and for controlled scientific experiments. Long-term experiments are needed to ensure sustainable biomass production. Water temperature was found to be a major factor affecting sponge growth. The 25 °C (Warm) treatment was found to be optimal, with a positive growth rate slope throughout the experiment. In contrast, in the 31 °C (Hot) treatment, the slope of the growth rate was negative (the sponges shrank). A balance between a high growth rate and higher water temperature is required to culture sponges with thermally stable collagen. During the experiment, the ambient treatment started at 19 °C and increased to 25 °C. As the ambient temperature increased from 18 to 25 °C, the growth rate of sponges in that treatment grew closer to that of the Warm (25 °C) treatment (Figure 2). This result further supports the conclusion that water temperature has a positive impact on sponge growth rate. 

It was found that the sponge *Chondrosia reniformis*, as a biotechnologically important source of collagen, is highly fit for culture, is amenable to handling and manipulations (both in sea and land-based systems), has high survivorship and a high growth rate, and seems to produce highly thermally stable collagen. 

## 4. Materials and Methods

### 4.1. Research Sites

**Figure 4:**
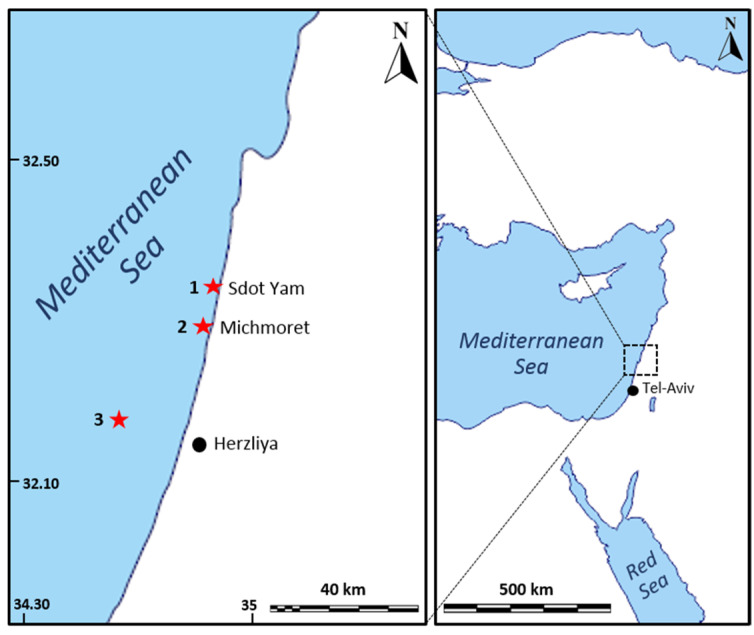
Research and collection site map. 1—shallow collection site at Sdot-Yam beach; 2—research site next to Michmoret beach; 3—mesophotic collection site 15 km off Herzliya’s coast.

The sea-based culture system experiment was conducted in the East Mediterranean Sea, off Israel’s Michmoret shore, at a depth of 10 m (32.40090° N, 034.86192° E, WGS-84; Figure 4). For this experiment, sponges were collected from shallow and mesophotic habitats (Table 1) and transplanted to a shallow rocky habitat where *C. reniformis* grows naturally. The mesophotic site is 15 Km off the coast of Herzliya. It is a sunken sandstone ridge at a 100–120 m depth (32.17710° N, 034.63306° E, WGS-84). The land-based culture system experiment was conducted at Ramot-Yam mariculture center, Michmoret, in an open seawater system.

### 4.2. Sponge Collection

**Figure 5:**
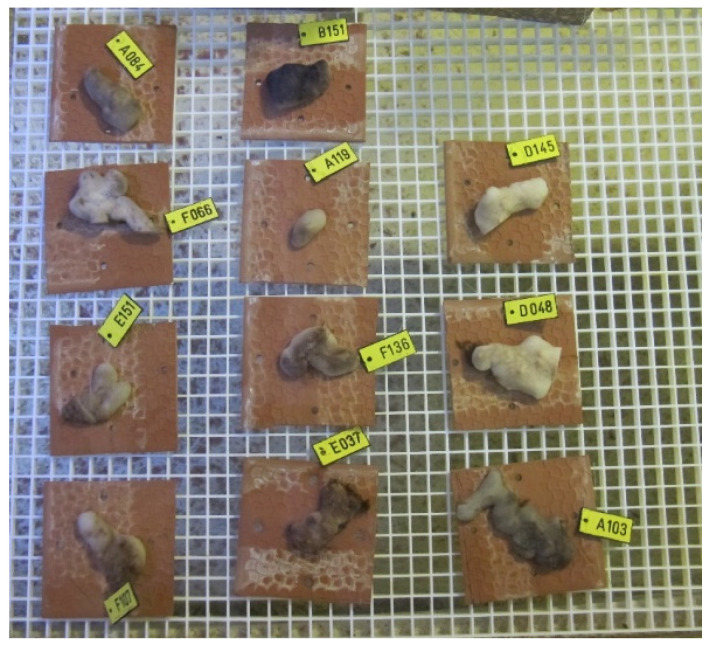
*Chondrosia reniformis* individuals attached to numbered clay tiles in a flow-through seawater system, before transplantation to the sea-based mariculture experiment site.

Sponges for the sea-based culture system were collected from two sites along the Israeli Mediterranean Sea between February and March 2019 (Figure 5 and Table 1). All sponges were collected with a permit (2019/42325) from the Israel Nature and National Parks Protection Authority.

The sponges from the mesophotic site were collected by a Remotely Operated Vehicle (ROV), ECA-Robotics H800, onboard the research vessel “Mediterranean Explorer”. This ROV is equipped with a five-function-manipulator and a full high-definition camera. Seventeen sponges were collected at ~100 m depth and brought to the surface in a collection basket, then instantly put in a seawater flow tank. At the shallow site, 62 sponges were collected at 2–6 m depth by SCUBA diving and brought to the surface in plastic bags. The sponges were cut from the rocks with a knife, leaving at least 50% of the individual connected to the substrate for regeneration. The explant size ranged from 10 to 15 cm^2^ with an average thickness of 1.5 cm. Specimens for the sea-based culture experiment and the land-based culture experiment were taken to Ramot-Yam mariculture center in Michmoret. They were attached to 10 × 10 × 1.5 cm clay and Perspex tiles, respectively, with a simple fishing line knot that went through two small holes in the tiles. All the sponges attached themselves naturally to the substrate within two weeks, some after three days (Figure 5). Every tile was marked with a number and put in an aquarium with flowing seawater system, until transplantation at the research site. Thirteen excess sponges were collected as backup, but not used in any experiment; they were returned to their natural habitat after the end of the experiments. Throughout the process, all the sponges were not exposed to air. 

### 4.3. Sea-Based Mariculture System Experiment 

**Figure 6:**
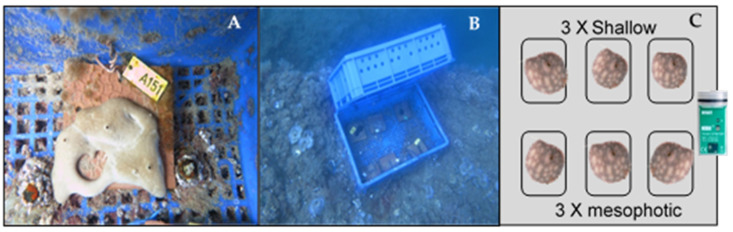
Sea-based mariculture system experiment. Each cage contains three *C. reniformis* individuals from the shallow habitat, three from the mesophotic habitat, and a data logger (HOBO Pendant^®^ Temperature/Light Data Logger). (**A**)—picture of a sponge attached to a tile connected inside the cage; (**B**)—A picture of an entire cage in the experiment site; (**C**)—A schematic drawing of the cage content.

The sea-based experiment was conducted between May 2019 and January 2020. In this setup, the growth of sponge transplants taken from two different habitats (mesophotic and shallow water) was tested. Before the experiment, mesophotic sponges grew in water temperatures of 16–20 °C, while shallow water sponges naturally grew in water temperatures of 17–31 °C. The sea-based system was constructed of plastic cages (55 × 15 × 29 cm) connected to the rocky terrain (10 m deep) with a pneumatic driller and metal bolts. The cages were constructed of two perforated plastic crates that were connected together to create low illumination and water flow conditions, mimicking the conditions of the sponge’s natural habitat on rocky shelves [47]. A data logger (HOBO Pendant^®^) was placed inside and outside the cages to log water temperature and illumination. Following the acclimation process described above, the sponges and tiles were taken by SCUBA diving and transplanted onto the sea-based system. There they were attached to five plastic cages. Six sponges were randomly transplanted in each cage, three from each original habitat (Figure 6). Overall, there were five cages, each containing six sponges, resulting in a total of thirty sponges in the experiment (*n* = 30). The cages were cleaned from fowling organisms every two weeks to ensure sufficient water flow and to lower the potential competition with other filter-feeders. 

We used the surface area of the sponges as a proxy for their size, as the thickness (height) of these sponges changes minimally between individuals, and the surface area was previously found to be strongly correlated with wet weight and volume [33]. On the semimonthly dives, each sponge was photographed with a Canon PowerShot G16 camera from a top view, to enable measurements of its surface area. The photos were analyzed with ImageJ^®^ software. The size of the tiles was used as a scale. The surface area (A) was calculated with a pixel-counting function [48]. Each individual’s surface area at a given time was compared to the surface area of the same individual at the start of the experiment to calculate the percent relative growth (or shrinkage):

$$Growth\left(\text{\%}\right)=\left(\frac{A_{1}\;-\;A_{0}}{A_{0}}\right)\times 100$$

### 4.4. Land-Based Culture Experiment 

The land-based culture experiment was conducted between February and June 2020, for 101 days, including acclimation. This experiment was designed to examine the effect of water temperature on *C. reniformis’s* growth rate in a controlled environment. For this setup, 42 sponges were collected from Sdot-Yam (Shallow habitat only) (Figure 4; Table 1) in February when the water temperature was 18 °C. Thirty-six sponges (*n* = 36) were used in the experiment, divided into three treatments and three aquaria in each treatment. The sponges were attached to black Plexiglas plates (10 × 10 × 0.5 cm) and placed in the flow-through seawater culture system, designed and built for this experiment. The black color of the plates was intended to create high contrast between the sponges and the plates for the picture analysis step. The sponges were connected to the plates and acclimated to ambient sea temperature for two weeks to allow regeneration and natural attachment. The experiment started when all sponges were healthy and attached to the plates. 

### 4.5. Experimental Design

**Figure 7:**
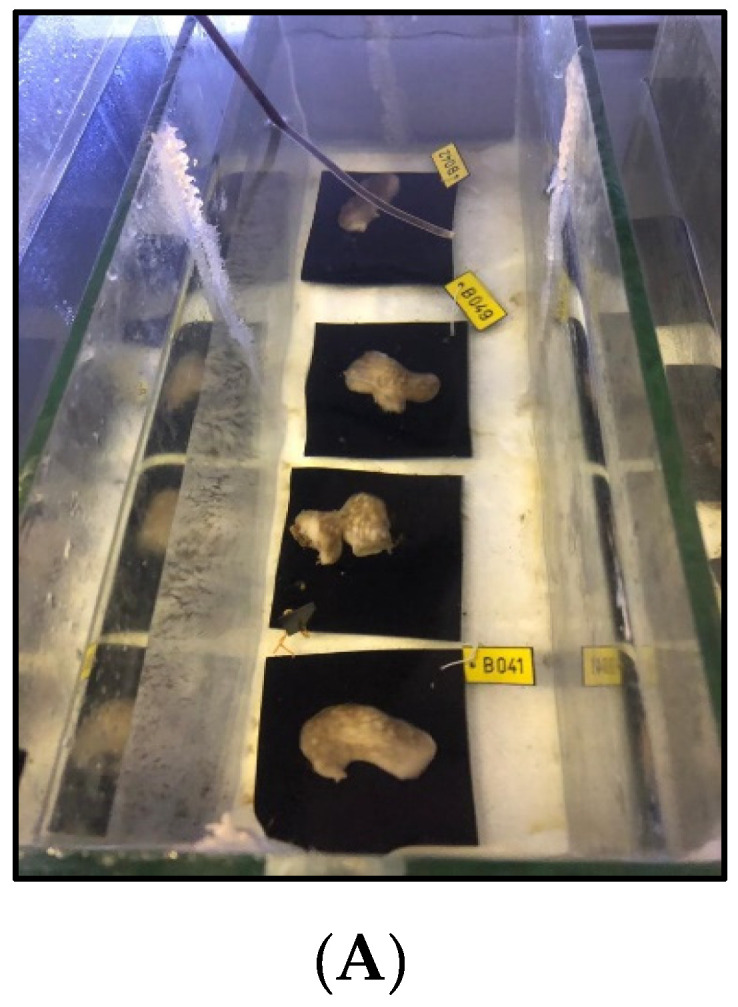

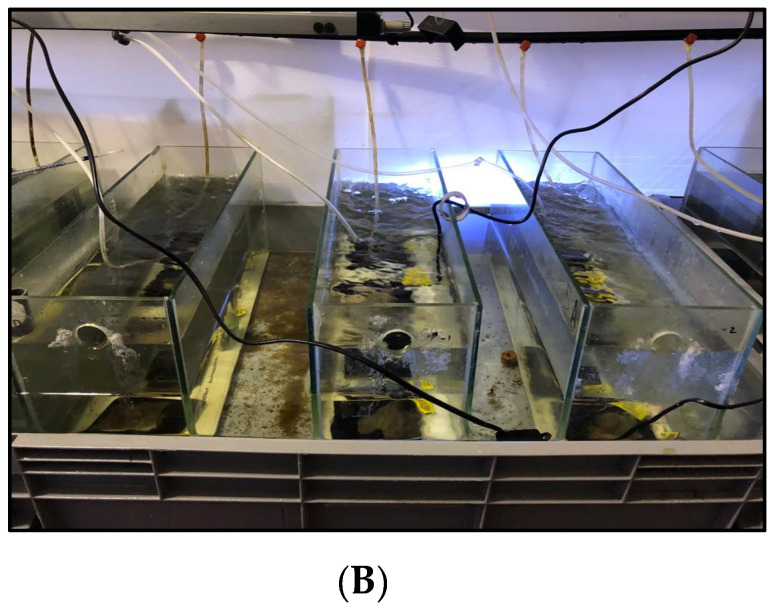
Land-based culture system. (**A**)—Four *C. reniformis* individuals attached to black Perspex tiles in one of the experiment’s aquaria. (**B**)—A plastic basin with three aquaria in the experiment system.

Three treatments were included in this experiment. In two of them, the water temperature was constant throughout the experiment, Hot treatment (31 °C), and Warm treatment (25 °C). These temperature values were based on the preceding sea-based experiment in which an optimal sponge growth rate occurred at a water temperature of 24–26 °C. The third treatment was the ambient local Mediterranean coastal (shallow) water temperature, which changed during the experiment, and served as a control. Every treatment included three glass aquaria (31 × 50 × 16 cm) placed in a plastic container (60 × 80 × 32 cm), used as a basin. Each aquarium contained four sponges, supplied with flowing seawater (sand filtered; flow speed: 170 mL/min) and an airflow tube (Figure 7). In the High-water temperature treatment, two heaters of 400 w (Newa therm vtx400^©^) and a simple water pump (320 L/hour) were placed in the plastic basin. The heaters were connected and controlled by a sensor and thermostat (Dixell^©^) with an accuracy of ±0.1 °C. The warm temperature treatment included only one heater. The basin’s water was heated to the desired temperature and stirred by the pumps to achieve a uniform temperature in the basin to heat the aquaria water. The water temperature in the basin was set to 1.5 °C higher than the desired water temperature in the aquaria, as determined by a preliminary experiment. In the ambient treatment, there were no heaters at all, and only one water pump was included. Each aquarium received a separate inflow of seawater. The overflow of each aquarium spilled to the basin of the specific treatment and heated it to the desired temperature, and thus, heated the water in the experiments’ aquaria. 

In this experiment, the temperature factor was isolated from all the other seasonal factors that were part of the sea-based culture experiment. The seawater was filtered, and the sponges received identical nutrition and illumination conditions. Therefore, the only changing variable between the treatments was the water temperature. The sponges were divided equally and randomly between the treatments (12 sponges in each treatment, four sponges in every aquarium) (Figure 7). A data logger (HOBO^©^) was placed in every aquarium to monitor the water temperature. Uniform and limited light conditions were kept throughout the experiment. Twice a day, the sponges were fed with 5 g powder of the cyanobacterium Spirulina (Seakura^©^) and suspended in 10 mL seawater, which were equally divided between aquaria. The acclimation started at 18 °C, and the temperature was raised by 1 °C every two days until reaching the desired temperature. The sponges were photographed once a week (canon power shot G16). The photos were then analyzed with ImageJ^®^ software to determine the surface area of the sponges, as described in the “Sea-Based Mariculture System Experiment” section.

### 4.6. Statistical Analysis

Statistical analyses were conducted using the stats package in R software (R core team 2020). The normality of the data and equality of variances were tested with a Shapiro-Wilk normality test and Levene’s test, respectively. The data of the sea-based mariculture system did not distribute normally. Therefore, the Mann–Whitney–Wilcoxon non-parametric test was used. The test examined differences in growth rates between sponges from two different habitats (*n* = 15 for each group). The growth rate of every sponge was calculated. Linear regression of the relative size and experiment time was conducted with LmerTest package [49], and the slope (growth rate) was determined for each individual sponge. In the land-based mariculture system, the differences in growth rates between treatments (*n* = 12 in each) were examined with a linear mixed model in order to include the aquaria as a random effect. Graphs were produced with the ggplot2 package (H. Wickham) and Microsoft Excel (2010). All tests were calculated with a significance level of α = 0.05, and the results were reported ± standard deviation (SD).

## 5. Conclusions

*Chondrosia renformis* are culturable. We raised *C. reniformis* both at sea and inland with 100% survivability. They even endured transplantation from the mesophotic depth to a shallow location. The method used prevented *C. reniformis* from “escaping” the culture system. Sponges originally from the shallow warmer habitat grew faster and larger, probably due to heat adaptability. This observation implies that they are better suited to providing a thermally stable collagen. We observed a seasonal effect on the *C. reniformis* growth rate. It was found that this sponge grows faster and larger at 26 °C water temperature. Further and long-term experiments are needed to ensure sustainable biomass production.

## Figures and Tables

**Table 1:** *Chondrosia reniformis* collection sites. Coordinates, depth, number of specimens, and date.

Collection Site	Coordinates	Depth (m)	Number of Specimens	Collection Date	Collected with	Collected for
**Mesophotic site off Herzliya’s coast**	32.17710° N, 34.63306° E	~100	9	11 February 2019	ROV	Sea-based mariculture system
			8	13 February 2019		
**Shallow site off Sdot-Yam coast**	32.40090° N, 34.86192° E	2–6	20	8 March 2019	SCUBA	Land-based mariculture system
			2	28 February 2020		

**Figure A1:**
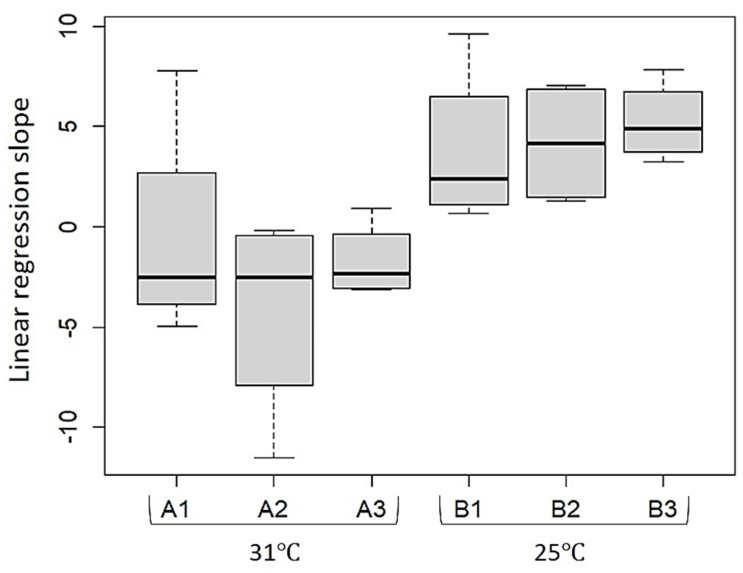
Linear regression slope of *C. reniformis* growth rate by aquaria in land-based culture system experiment. Box plots for treatments’ aquaria. *n* = 4 in each aquarium; line inside the box = median of regression slopes in each aquarium; box = 25–75 percentiles; lines outside the box = min and max values; A1–A3 = Hot treatment aquaria; B1–B3 = Warm temperature treatment aquaria; Statistical significant difference (Factorial Anova, sum sq. = 256.65, *F*-value = 18.169, *p* < 0.05).

## Data Availability

Data available in a publicly accessible repository. The data presented in this study are openly available in [FigShare] at [https://doi.org/10.6084/m9.figshare.17082071.v1, accessed on 19 October 2021], reference number [50].

## Appendix A

**Table A1:** *p*-values of mean growth rate differences between groups in the sea-based mariculture system experiment.

Measurement Date	*p*-Value
21 May 2019	0.744
4 June 2019	0.137
24 June 2019	<0.0001
12 July 2019	0.0008
25 July 2019	0.0001
8 August 2019	0.0128
26 August 2019	0.0086
12 September 2019	0.0235
26 September 2019	0.0042
10 October 2019	0.0128
7 November 2019	0.0086
21 November 2019	0.0235
21 December 2019	0.0975

**Table A2:** Water temperature of the sea-based mariculture system during the experiment.

Measurement Date	Water Temperature (℃)
6 May 2019	21
21 May 2019	22
4 June 2019	25
24 June 2019	27
12 July 2019	29
25 July 2019	29
8 August 2019	30
26 August 2019	30
12 September 2019	30
26 September 2019	29
10 October 2019	28
7 November 2019	25
21 November 2019	22
21 December 2019	20
